# Daily Changes in Channel Occupancy in the 868 MHz ISM Band

**DOI:** 10.3390/s22249928

**Published:** 2022-12-16

**Authors:** Sebastian Kozłowski, Krzysztof Kurek

**Affiliations:** Institute of Radioelectronics and Multimedia Technology, Warsaw University of Technology, ul. Nowowiejska 15/19, 00-665 Warsaw, Poland

**Keywords:** IoT, channel occupancy, ISM band

## Abstract

Due to the rapid development of Internet of Things (IoT) systems operating in industrial, scientific and medical (ISM) frequency bands, many researchers have attempted to determine the amount of interference that can be expected in such systems. The basic information required for this purpose is the current occupancy of frequency channels in various geographical locations. It is known that the occupancy measurement must last long enough to allow for the detection of low duty cycle transmissions. In this paper, it is shown that fulfilling only this criterion may lead to unreliable results being obtained. In two measurement campaigns performed in two different locations, the occupancy of a selected sub-band in the 868 MHz ISM band was determined on the basis of two hour-long observations repeated several times a day. During a typical day, the ratio of the maximum and the minimum result depended on the location and reached a value of eight; however, on one day, a period of abnormally high channel usage reaching 65% was observed in the location in which typical values did not exceed 1%.

## 1. Introduction

The distinguishing feature of IoT systems is that they generate very low traffic. A typical IoT wireless node transmits short data portions at long time intervals. The duty cycle of such a transmission can be as low as 0.1% [1,2], which corresponds to 3.6 s available for transmission per hour. For this reason, a number of IoT standards, e.g., LoRa or SigFox, allow the use of unlicensed ISM bands with no time synchronization between the nodes of a single network or with other users of the given frequency channel. Access to the wireless medium is obtained in a random manner based on the assumption that low medium occupancy assures a low access collision probability.

This approach works well if only a few wireless nodes operate in the same band and in the same geographical location. However, one can observe that the number of IoT devices is constantly and rapidly growing, as well as the number of other users of unlicensed bands not classified as IoT. As examples, one can mention Wi-Fi and Bluetooth devices in the 2.4 GHz ISM band, as well as automatic gates, car keys, weather stations, alarms, and radio-frequency identification (RFID) tags in the 433 MHz and 868 MHz ISM bands. Under such circumstances, deploying a new, or expanding an existing, wireless network may face a problem of frequent access collisions. For this reason, it is justified to make efforts to monitor the current occupancy of an electromagnetic spectrum on a regular basis.

A number of researchers have presented measurements of the total wireless medium occupancy in various frequency bands intended to be used by different systems. Much attention has been paid to wireless local area networks (WLANs) operating either in the 2.4 GHz ISM band [3,4,5,6] or the 5 GHz ISM band [7,8,9]. FM broadcasting was analyzed in terms of spectrum usage in the frequency range of 88–108 MHz in [10]. Wideband measurements, covering multiple bands, were reported in [11,12,13,14,15,16].

This paper focuses on the 868 MHz ISM band. The occupancy of this particular part of the radio spectrum was the subject of [2,17,18,19,20]. In [2,17], the traffic intensity and resulting interferences were analyzed on the basis of measurements taken in five different locations. A single location was observed for two hours to capture at least one cycle of the expected low duty cycle devices. For the same reason, a two hour-long observation time was adopted in [18]. In [19], the usage of the sub-band intended for RFID tags was investigated. A single observation time was 10 min per location since the transmission was expected to be repeated every 0.5–5 s in this particular sub-band.

In contrast to previous works, the authors of [20] presented results obtained during both short-term and long-term campaigns. The short-term measurement lasted for two hours, whereas during the long-term measurement over two thousand 5 min-long observations were taken, which corresponded to a total measurement time of 7 days. The time changes of the spectrum occupancy were not the main focus of the paper and were not deeply analyzed; however, it was indicated that the occupancy may either be constant or may change rapidly, depending on the particular sub-band. 

From the above survey of the literature, one can conclude that a two hour-long observation time is widely considered to be sufficiently long for measurements in the band of interest. On the other hand, in [20] it was shown that the occupancy may change, so taking a two hour-long measurement only once per location may lead to unreliable results. In this paper, daily changes in the channel occupancy are presented on the example of a selected sub-band in two out of the six locations from [18]. The measurement procedure presented therein was modified in the sense that, instead of making a single observation per location, a number of consecutive observations were made in order to determine how much their results may vary.

## 2. Measurement Methodology

### 2.1. Measurement Set-Up and Data Processing

**Figure 1:**
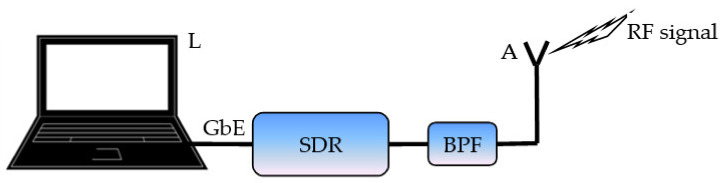
A block diagram of the measurement set-up.

The measurement set-up used, presented in detail in [18], is shown in Figure 1 in the form of a block diagram. Its main part was a software-defined radio (SDR) module, NI USRP 2932, communicating with a laptop computer (L) via a Gigabit Ethernet (GbE) connection. The laptop operated as a signal processing device for the entire set-up. The radio frequency (RF) signal was received using a simple vertically polarized monopole antenna (A) and was provided to the input of the SDR module via a band-pass filter (BPF) which, at least partially, attenuated signals outside of the band under test which could affect the measurements.

The dedicated software running on the laptop analyzed the baseband signal from the SDR module to extract bursts, i.e., parts of the received signal considered to be non-noise. It was assumed that a burst starts when a sample with a real or imaginary component exceeding a predefined threshold is detected, and ends when no such sample is detected for a predefined time period. Gaps shorter than this predefined time period (0.5 ms) were ignored to prevent the algorithm from detecting particular symbols as separate bursts in the cases of transmission schemes such as on-off keying, pulse width or pulse position modulation. 

Once a burst was detected, a number of its characteristics, including length and average power, were logged in a text file. It should be noted that the received signal itself was not recorded; thus, the frequency channel under test could be observed for a very long time without generating an enormous amount of data. 

After the observation was completed, the log file contained information about all detected bursts. The channel occupancy was computed in the post-processing stage as an aggregated length of the bursts relative to the total observation time, and was conveniently expressed in percent. For the investigation presented in this paper, a single observation was assumed to last two hours or until one million bursts were logged. As stated in many previous works, this is considered to be sufficient to obtain reliable results for channels in which low duty cycle devices are operating.

### 2.2. Measurement Locations and Frequency Band of Interest

**Figure 2:**
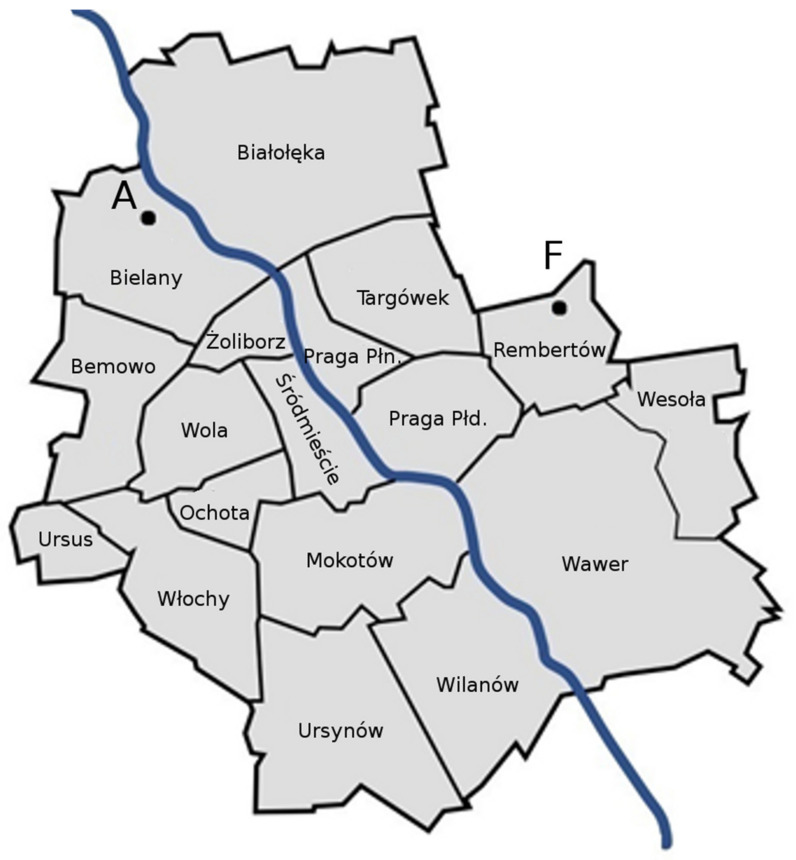
Measurement locations on a map of Warsaw.

The measurements were carried out from January to March 2022 in two apartments located in residential districts of Warsaw, Poland, in the 868.0–868.6 MHz frequency range. In correspondence with the nomenclature used in [18], the measurements locations were referred to as A and F (Figure 2).

## 3. Results and Discussion

### 3.1. Variations of the Channel Occupancy

**Figure 3:**
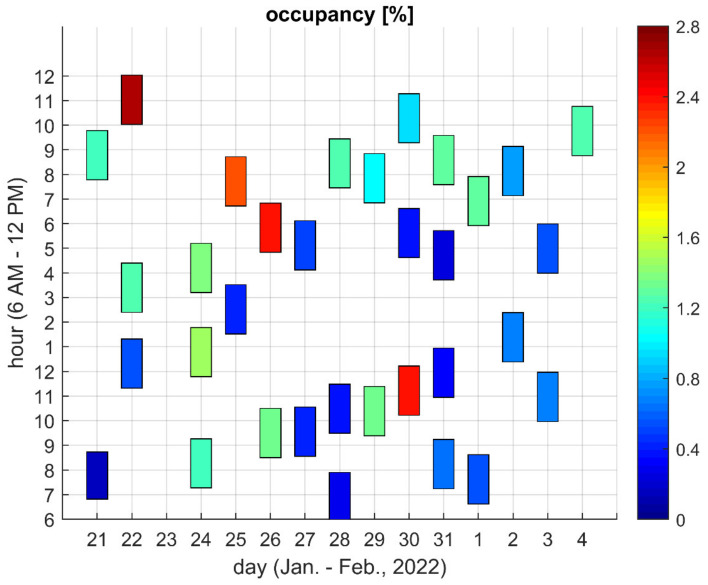
Channel occupancy for Location A.

**Figure 4:**
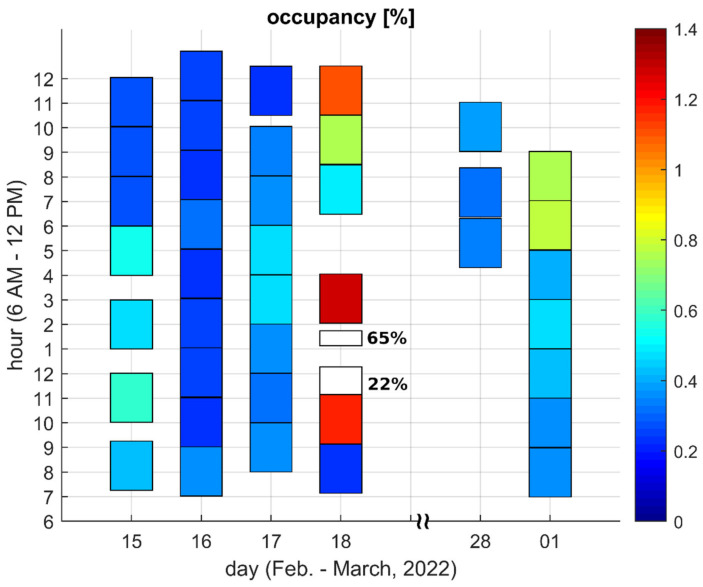
Channel occupancy for Location F.

Figure 3 and Figure 4 present measured channel occupancy values for Locations A and F, respectively. Each patch visible in the figures corresponds to a single observation. The position and height of the patch indicate the observation time, whereas the occupancy value is indicated by the patch color. The heights of most of the patches correspond to the two hour-period, with the exception of two patches in Figure 4, which are noticeably shorter. These two patches correspond to experiments where the criterion of 1 million bursts was fulfilled before the nominal observation period ended. 

**Figure 5:**
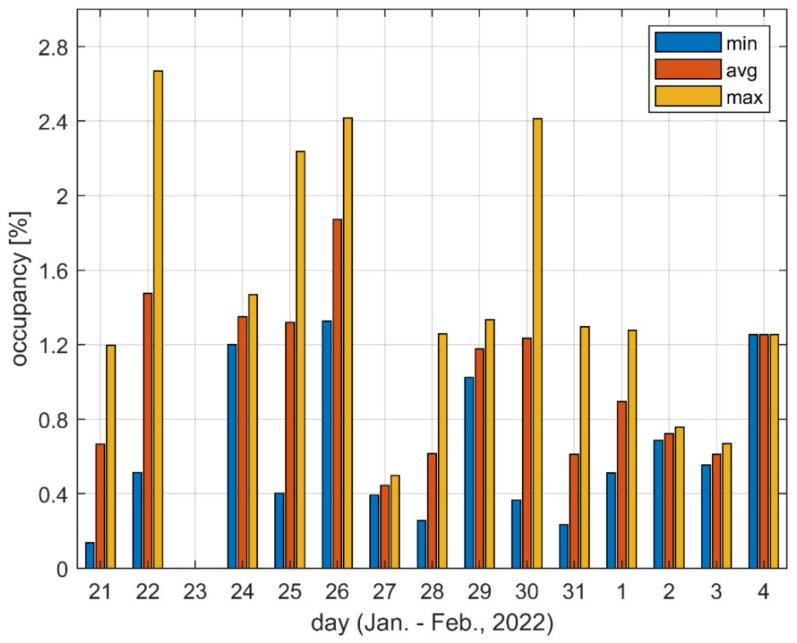
Minimum, average and maximum observed channel occupancy values for Location A.

**Figure 6:**
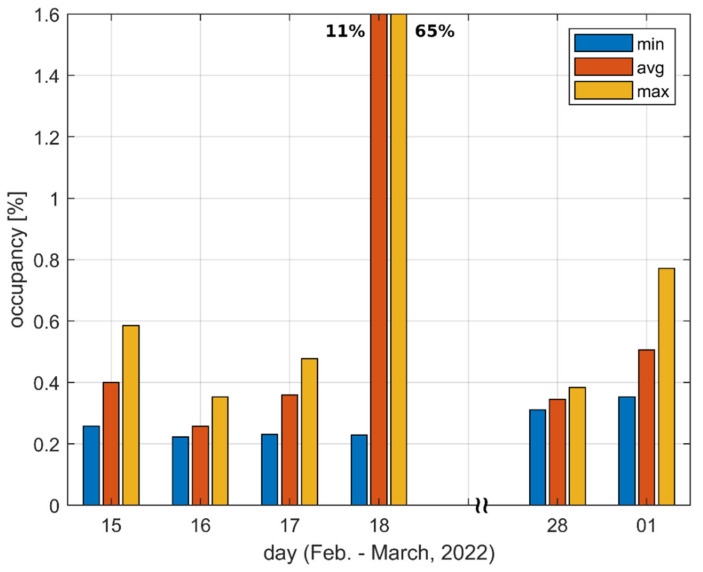
Minimum, average and maximum observed channel occupancy values for Location F.

Figure 5 and Figure 6 present the minimum, average, and maximum occupancy values observed during a given day. The minimum and maximum values correspond to the darkest and lightest patches, respectively, presented in Figure 3 or Figure 4 for a given day and a given location. Similarly, the average was taken over the values represented by all patches for a given day and a given location. In other words, minimum, maximum and average values are taken over particular columns of patches visible in Figure 3 and Figure 4.

The figures show that in Location A, the results are below 3% and in Location F, they are below 1% with the exception of one day which will be discussed later.

The obtained results showed no obvious relationship between the occupancy value and the time of day. Relatively higher values were equally likely to occur in the morning, afternoon and late evening. In Location A, no day could be claimed as being unusual in terms of channel usage. On the contrary, in Location F, the channel was untypically busy on 18 February from approximately 11 AM to 2 PM, when an occupancy value as high as 65% was detected. However, since the logged data included limited information about detected bursts, it was not possible to determine whether the occupancy resulted from an intended wireless transmission or from unintended interference from some non-communication electronic device used on a single occasion in the vicinity of the measurement site. Anyway, it is irrelevant since, regardless of the origin of the detected emissions, they prevented or at least limited communication in the channel under test.

Figure 5 shows that the ratio of maximum and minimum occupancy values determined during a day frequently equaled five or more in Location A (it equaled eight on 21 January). Figure 6 shows that this same ratio for Location F was usually equal to around two. Accordingly, it was proven that when only a single measurement was taken in a given location, the result may be either underestimated, or may correspond to the worst conditions present in the channel only for a fraction of time. 

### 3.2. Comparison with Previous Work

With regard to [18], one can notice that during the measurement campaign described therein (carried out in 2020), the 868.0–868.6 MHz frequency range was unoccupied in Location A; thus, no comparison with the present results can be made. As far as Location F is concerned, the occupancy was determined to be approx. 0.3%. This corresponds well with the present results, in which the average value ranged from 0.25 to 0.5%. However, the previous campaign did not show that the occupancy may occasionally reach 0.6–0.8%. It also provided no indication that the occupancy may even reach 65%. 

## 4. Conclusions

This paper demonstrated that a single observation may not provide reliable information about the channel occupancy. In one of the measurement locations, the ratio of the maximum and the minimum result did not exceed two, with one exception which requires a separate analysis. Taking into consideration that the maximum itself did not exceed 1%, it can be assumed that the observed variations would have a moderate impact on the systems operating in the band of interest. On the other hand, in the second location, the abovementioned ratio ranged typically from two to five, although a value of eight was also noted. This means that, when a single observation is taken, the channel occupancy is known with an accuracy slightly better than an order of magnitude. Given that the maximum value for this case did not exceed 3%, the channel under test still could not be considered as busy. However, if such variations in the measurement results continue in the future, when the number of wireless devices will be much greater, the unpredictable and improper operation of these devices may occur due to mutual interferences not predicted because of the significant underestimation of the channel occupancy. To avoid this problem, one should repeat the measurement several times or, equivalently, the observation time should be appropriately extended in relation to the value that is considered to be sufficient to catch low duty cycle transmissions.

This study also revealed that an abnormally high channel occupancy value may be occasionally measured. By taking the result of such a measurement without any validation, one can drastically overestimate the actual average usage of a channel.

This paper only used the lengths of detected bursts for the analysis. The measurement set-up logged some other burst characteristics (see [18] for more detail), but they are still not sufficient to allow the system which the given burst originates from to be identified. Developing the set-up towards the removal of this limitation will be the subject of future work. 

## Data Availability

The logs used for the presented analysis are available from the authors upon reasonable request.
